# Singlet oxygen initiates a plastid signal controlling photosynthetic gene expression

**DOI:** 10.1111/nph.14223

**Published:** 2016-10-13

**Authors:** Mike T. Page, Alex C. McCormac, Alison G. Smith, Matthew J. Terry

**Affiliations:** ^1^ Biological SciencesUniversity of SouthamptonSouthamptonSO17 1BJUK; ^2^ Department of Plant SciencesUniversity of CambridgeCambridgeCB2 3EAUK; ^3^ Institute for Life SciencesUniversity of SouthamptonSouthamptonSO17 1BJUK

**Keywords:** chlorophyll synthesis, chloroplast development, photosynthesis, regulation of gene expression, retrograde signalling, singlet oxygen (^1^O_2_), tetrapyrroles

## Abstract

- Retrograde signals from the plastid regulate photosynthesis‐associated nuclear genes and are essential to successful chloroplast biogenesis. One model is that a positive haem‐related signal promotes photosynthetic gene expression in a pathway that is abolished by the herbicide norflurazon. Far‐red light (FR) pretreatment and transfer to white light also results in plastid damage and loss of photosynthetic gene expression. Here, we investigated whether norflurazon and FR pretreatment affect the same retrograde signal.
- We used transcriptome analysis and real‐time reverse transcription−polymerase chain reaction (RT‐PCR) to analyse the effects of these treatments on nuclear gene expression in various Arabidopsis (*Arabidopsis thaliana*) retrograde signalling mutants.
- Results showed that the two treatments inhibited largely different nuclear gene sets, suggesting that they affected different retrograde signals. Moreover, FR pretreatment resulted in singlet oxygen (^1^O_2_) production and a rapid inhibition of photosynthetic gene expression. This inhibition was partially blocked in the *executer1executer2* mutant, which is impaired in ^1^O_2_ signalling.
- Our data support a new model in which a ^1^O_2_ retrograde signal, generated by chlorophyll precursors, inhibits expression of key photosynthetic and chlorophyll synthesis genes to prevent photo‐oxidative damage during de‐etiolation. Such a signal would provide a counterbalance to the positive haem‐related signal to fine tune regulation of chloroplast biogenesis.

## Introduction

Communication between the nucleus and plastids (most notably the chloroplasts) is crucial for plant cell function. The nucleus maintains control over most aspects of chloroplast development and function (Jarvis & López‐Juez, 2013), but it has been recognized for over three decades that chloroplasts also exert a retrograde influence on nuclear gene expression (Bradbeer *et al*., 1979). Many signalling molecules have been implicated in plastid‐to‐nucleus communication (Kleine *et al*., 2009; Pfannschmidt, 2010; Chi *et al*., 2013; Chan *et al*., 2016), with the best characterized operating in mature plants in response to a range of stresses (Estavillo *et al*., 2011; Xiao *et al*., 2012). Reactive oxygen species (ROS) have also been shown to be important, with chloroplast‐derived superoxide, hydrogen peroxide (H_2_O_2_) and singlet oxygen (^1^O_2_) all able to regulate nuclear gene expression (Galvez‐Valdivieso & Mullineaux, 2010). In particular, extensive characterization of the *fluorescent* (*flu*) mutant of Arabidopsis (Meskauskiene *et al*., 2001) has revealed an important role for chloroplast‐derived ^1^O_2_ in mediating stress acclimation and cell death responses (Kim *et al*., 2012; Kim & Apel, 2013). In this experimental system, ^1^O_2_ is generated by photo‐excitation of the chlorophyll precursor protochlorophyllide (Pchlide), which accumulates in dark‐grown *flu* seedlings (op den Camp *et al*., 2003). The nature of this ^1^O_2_ signalling pathway is unknown, but, as ^1^O_2_ signalling has a short half‐life, signals would need to originate within the chloroplast. Some possible components have been identified, the most prominent of which are EXECUTER1 (EX1; Wagner *et al*., 2004) and EX2 (Lee *et al*., 2007). These related, chloroplast‐localized proteins are both required for *flu*‐mediated induction of ^1^O_2_‐regulated genes (Lee *et al*., 2007). Recently, Woodson *et al*. (2015) also identified a protoporphyrin IX‐induced, ^1^O_2_‐signalling pathway leading to ubiquitin‐mediated degradation of damaged chloroplasts that may be important in stress adaptation.

In contrast to signals involved in environmental stress responses, signals mediating retrograde signalling in seedlings during chloroplast biogenesis have proved elusive. Many of the early studies on retrograde signalling demonstrated a catastrophic loss of nuclear gene expression either in mutant seedlings lacking functional chloroplasts (Harpster *et al*., 1984; Hess *et al*., 1994) or in wild‐type (WT) seedlings subjected to chemical treatments that disrupt chloroplast function (Mayfield & Taylor, 1984; Oelmüller *et al*., 1986). The most commonly used treatment, the herbicide norflurazon (NF), inhibits carotenoid synthesis, causing plastid‐specific photo‐oxidative damage and resulting in a severe reduction in expression of photosynthetic genes, exemplified by *LIGHT HARVESTING CHLOROPHYLL A/B BINDING PROTEIN 1.2* (*LHCB1.2*) encoding a light‐harvesting chlorophyll‐binding protein (Strand *et al*., 2003; Koussevitzky *et al*., 2007; Moulin *et al*., 2008; Aluru *et al*., 2009). What we know about this biogenic retrograde signal has come mostly from the identification of *genomes uncoupled* (*gun*) mutants in Arabidopsis that retain partial *LHCB1.2* expression in NF‐bleached seedlings (Susek *et al*., 1993; Mochizuki *et al*., 2001; Larkin *et al*., 2003; Koussevitzky *et al*., 2007; Woodson *et al*., 2011). Of the original five *gun* mutants identified, the *gun2, gun3, gun4* and *gun5* mutations are all in genes involved in tetrapyrrole synthesis (Mochizuki *et al*., 2001; Larkin *et al*., 2003), with the *gun5* mutation residing in the H subunit of magnesium (Mg) ‐chelatase (CHLH) and resulting in reduced synthesis of Mg‐porphyrins (Mochizuki *et al*., 2001). GUN1 is a chloroplast‐localized pentatricopeptide‐repeat protein that is predicted to have nucleotide‐binding activity (Koussevitzky *et al*., 2007) and, in contrast to *gun2‐gun5*,* gun1* can also rescue nuclear gene expression under other conditions affecting chloroplast development, such as treatment with lincomycin, an inhibitor of plastid translation (Gray *et al*., 2003; Koussevitzky *et al*., 2007). Initial analysis of the tetrapyrrole‐related *gun* mutants led to the hypothesis that the tetrapyrrole Mg‐protoporphyrin IX, a chlorophyll biosynthesis intermediate, is a mobile retrograde signal (Strand *et al*., 2003). This was not supported by further biochemical and genetic studies (Mochizuki *et al*., 2008; Moulin *et al*., 2008) and instead a new model has been put forward in which a ferrochelatase1 (FC1)‐dependent, haem‐related signal acts positively to promote expression of nuclear photosynthesis genes (Woodson *et al*., 2011). However, a role for Mg‐protoporphyrin as an inhibitory plastid signal continues to be proposed (e.g. Kindgren *et al*., 2012).

A strong inhibition of nuclear gene expression is also observed following a pretreatment of Arabidopsis seedlings with far‐red light (FR) before transfer to white light (WL) (McCormac & Terry, 2002, 2004). Under FR, the phytochrome A photoreceptor (phyA) induces expression of nuclear‐encoded chloroplast proteins, but as FR cannot be utilized by the light‐dependent chlorophyll synthesis enzyme protochlorophyllide oxidoreductase (POR), chloroplast development is stalled (Barnes *et al*., 1996). Instead, accumulation of Pchlide and depletion of the POR proteins, which bind and buffer photosensitive Pchlide, result in severe photo‐oxidative damage to chloroplasts (Sperling *et al*., 1997; McCormac & Terry, 2004) and inhibition of nuclear gene expression (McCormac & Terry, 2002, 2004). Here, we tested whether NF and FR pretreatments target the same retrograde signal by measuring their impact on global gene expression. Our analysis shows that not only are the response profiles different, but the FR pretreatment identifies a previously undescribed pathway in which ^1^O_2_ mediates the inhibition of photosynthesis‐related nuclear genes. This novel inhibitory retrograde signalling pathway would provide a counterbalance to a positive haem‐related signal driving chloroplast biogenesis during seedling development.

## Materials and Methods

### Plant material and accessions

The WT Arabidopsis (*Arabidopsis thaliana* L.) line used in this study was Columbia (Col‐0). The single mutants *gun1* and *gun5* and the *gun1gun5* double mutant have been previously described (Vinti *et al*., 2000; Mochizuki *et al*., 2001), as has the *phyA* mutant (in the Col‐0 background) (see McCormac & Terry, 2002). The *ex1, ex2* and *ex1ex2* double mutants have been previously described (Wagner *et al*., 2004; Lee *et al*., 2007). Standard growth conditions (including growth medium and light sources) were as described previously (McCormac & Terry, 2002). Arabidopsis Genome Initiative accessions for genes mentioned in this study are given in Supporting Information Table S1.

### RNA extraction

Total RNA extraction was carried out as previously described (McCormac *et al*., 2001), but with the addition of a further purification step using the Qiagen RNeasy kit according to the manufacturer's instructions. Total RNA samples for reverse transcription−polymerase chain reaction (RT‐PCR) analysis were treated using the method described by Manning (1991) for the removal of polysaccharides. Polysaccharides were precipitated using 0.1 volumes of 1 M sodium acetate (NaOAc), pH 4.5, and 0.4 volumes of ethylene glycol monobutyl ether (2‐BE). The sample was incubated on ice for 30 min and centrifuged at 20 000 ***g*** for 10 min, and RNA precipitation from the supernatant was achieved by adding a further 0.6 volumes (with respect to the original RNA sample) 2‐BE, incubation for 30 min on ice and centrifugation at 20 000 ***g*** for 10 min. The pellet was washed consecutively with 40 mM NaOAc (pH 4.5) : 2‐BE (1 : 1), 70% ethanol (v/v) and 100% ethanol and air dried.

### Microarray analysis

For the FR pretreatment experiment, WT and *phyA* seedlings were grown on 1 × Murashige and Skoog (MS) salts without sucrose for 1 d in dark (D) followed by 2 d of continuous FR (or maintained for 2 d in D) before transfer to continuous WL for 1 d. Under these conditions, WT seedlings retain RNA and membrane integrity (McCormac & Terry, 2004). However, to ensure a full block‐of‐greening response in seedlings depleted of Pchlide, *gun1gun5* seedlings were grown for 3 d in FR before growth for 1 d in WL, with *gun1gun5* control seedlings receiving 3 d of D before 1 d of WL. For NF treatment, WT and *gun1gun5* seedlings were grown for 3 d in D followed by 3 d in WL on a medium containing 1 × MS salts and 1.5% (w/v) sucrose with or without 5 μM NF. RNA samples for each treatment were extracted from two fully independent experiments that were analysed separately (with the exception of *phyA* samples, which had one replicate). Microarrays were produced by the GARNet facility (University of Nottingham, Nottingham, UK) using 22K Affymetrix (Santa Clara, CA, USA) ATH1 Arabidopsis chips. Full microarray data sets are deposited in the National Center for Biotechnology Information (NCBI) GEO database (http://www.ncbi.nlm.nih.gov/gds; FR = GSE6169; NF = GSE5726). Analysis of the normalized data was conducted using Microsoft Excel and normalized signal data were filtered for a positive ‘transcript‐present’ score in both replicates of the WT control or treatment samples. Genes inhibited following an FR pretreatment were identified according to a consistent (i.e. both replicates) signal fold‐ratio of FR : D ≤ 0.5. Rescue of gene expression after an FR pretreatment in *gun1gun5* and *phyA* mutants compared with WT was calculated as the mutant treatment : control ratio divided by the WT treatment : control ratio, using a cut‐off of 1.5‐fold. Genes inhibited by NF were identified according to a consistent signal fold‐ratio of NF : control ≤ 0.5. A criterion of ≥ 1.5‐fold increase in NF‐treated *gun1gun5* seedlings compared with WT NF‐treated samples was used to identify *gun1gun5* rescued genes. For both NF and FR arrays, induced genes were identified according to a signal fold‐ratio of treatment : control ≥ 2.0 in both replicates. All further analysis of the microarray data, including comparisons with other microarray data sets, was performed in Microsoft Excel. Heat maps were generated using Multiexperiment Viewer (mev v.4.8.1; Saeed *et al*., 2003).

### Real‐time RT‐PCR

For direct comparison with microarray data using real‐time RT‐PCR, WT and *gun1gun5* seedlings were grown in the presence of NF or received an FR pretreatment, along with the respective controls, under the same conditions as described in the previous section for microarray analysis. In addition, the NF experiment was also carried out in the absence of sucrose. cDNA synthesis and real‐time PCR were carried out as described by McCormac & Terry (2004) and primer pairs are given in Table S1. Transcript abundance was calculated relative to *18S* rRNA within each sample. The real‐time RT‐PCR data for each treatment are expressed relative to the respective WT control samples and the signal values for the corresponding array data were normalized accordingly. For time‐course analyses, WT, *gun1* and *gun5* seedlings were grown for 1 d in D followed by 2 d in FR or kept for 3 d in D (± 5 μM NF) on medium without sucrose. All seedlings were transferred to WL at *t* = 0 and total RNA samples extracted at the times indicated. Transcript abundance was calculated relative to *18S* rRNA within each sample. For comparison of WT and the *ex* mutants, seedlings were grown for 1 d or 2 d in D followed by either 2 d in FR or 2 d in D (controls), and then transferred to WL for 24 h. Transcript abundance was calculated using real‐time RT‐PCR relative to *ACTIN DEPOLYMERIZING FACTOR 2* (*ADF2*) within each sample and confirmed using a second reference gene, *YELLOW LEAF SPECIFIC GENE 8* (*YLS8*). As shown in Fig. S1, all three reference genes give an equivalent response for the protocols used in this study.

### DanePy fluorescence quenching

Seedlings were grown with or without a 2‐d FR pretreatment or in D for 2 d with or without 5 μM NF and infiltrated with 50 mM KPO_4_ (pH 7.2) (1% v/v ethanol) containing 200 μM DanePy (a gift from Kalman Hideg, University of Pécs, Hungary) using a plastic syringe as described by Hideg *et al*. (2002). Twenty seedlings per treatment were infiltrated with 2 ml of solution and incubated under WL for 5 h. Fluorescence spectra of samples (excitation 330 nm) were measured using an F‐2000 spectrophotometer (Hitachi, Tokyo, Japan) and values were recorded for the emission maxima (532 nm); seedlings were removed from the solution before measurement.

### Imaging Singlet Oxygen Sensor Green fluorescence

Seedlings were grown for 2 d in the dark, followed by a 3‐d FR pretreatment. At 150 min before the end of the third day in FR, seedlings were immersed in a solution of 10 μM Singlet Oxygen Sensor Green (SOSG; ThermoFisher, Waltham, MA, USA) for 2 h in FR, then gently blotted dry and returned to their growth environment for 30 min. Seedlings were transferred to WL and excised cotyledons were imaged with fluorescence microscopy using a Zeiss Axioplan2 microscope (excitation = 470/40 nm; dichroic = 495 nm (LP); emission = 525/50 nm) with an integration time of 100 ms. Control seedlings either remained in the dark for 5 d or were imaged after FR without SOSG treatment to account for background fluorescence from the plant tissue. For the first time‐point (0 h, before transfer to WL), slides were prepared in the dark under a dim green safelight and maintained in the dark before imaging by wrapping in foil. All images were acquired using the same objective lens (×10), and intensity histograms were kept constant for all images shown. The SOSG signal for each sample was determined in Imagej (NIH, Bethesda, MD, USA) by assessing the signal averaged over the area of one cotyledon. Each data point represents the mean SOSG signal of three cotyledons from seedlings assayed in independent biological replicates. The same microscope settings were used to acquire all images.

### Pigment analysis

Chlorophyll and Pchlide was assayed for 20 seedlings as described in Stephenson & Terry (2008) and Stephenson *et al*. (2009), respectively.

## Results

### NF and FR pretreatments target different retrograde signals

**Figure 1:**
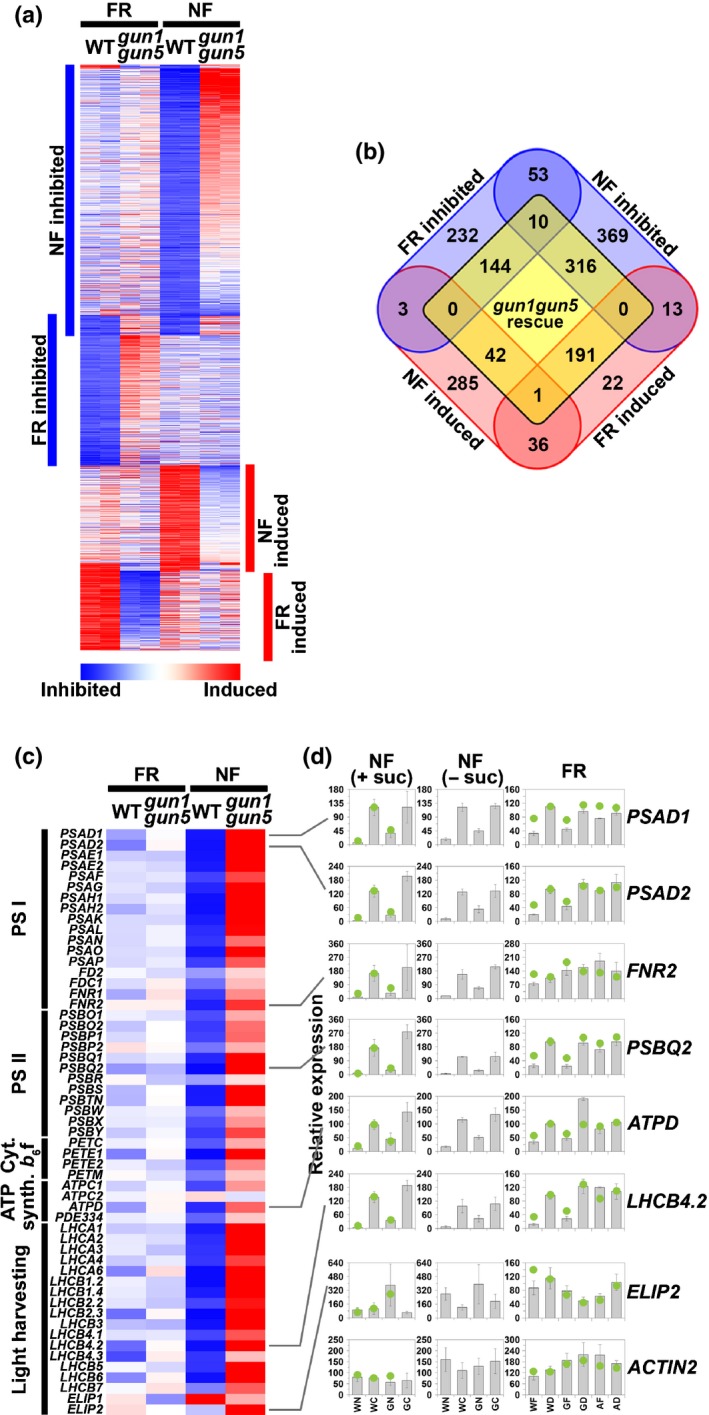
Analysis of the transcriptional response under white light of wild‐type (WT; Columbia (Col‐0)) and *gun1gun5 Arabidopsis thaliana* seedlings treated with either 5 μM norflurazon (NF) or a far‐red light (FR) pretreatment. (a) Heatmap depicting a gene cluster analysis of microarray data for nuclear genes inhibited (blue) or induced (red) at least two‐fold by either NF or FR pretreatment in WT in two independent biological replicates. For the FR *gun1gun5* column, expression is shown as the difference between treatment and controls in *gun1gun5* relative to the difference in WT. For the NF *gun1gun5* column, expression is shown as the difference between NF‐treated WT and *gun1gun5*. (b) Venn diagram demonstrating the number of genes inhibited (blue) or induced (red) at least two‐fold by NF or an FR pretreatment, and the number of genes rescued 1.5‐fold in *gun1gun5* (yellow). (c) Heat map depicting microarray analysis of inhibited (blue) or induced (red) photosynthesis‐related genes grouped by photosynthetic complex. Ratios are the mean of two independent experiments. Columns are represented as in (b). (d) Real‐time reverse transcription−polymerase chain reaction (RT‐PCR) analysis of representative photosynthesis‐related genes. For the NF experiments, WT and *gun1gun5* seedlings were grown in the presence (WN and GN) or absence (WC and GC) of 5 μM NF with and without 1.5% (w/v) sucrose. For the FR pretreatment experiments, WT,* gun1gun5* or *phytochrome A* (*phyA*) mutant seedlings were grown with a pretreatment of FR (WF, GF and AF, respectively) or kept in darkness (WD, GD and AD, respectively). Data shown are the mean ± SE (*n* = 3 (NF) or *n* = 7 (FR) independent experiments) with array data represented by green dots, normalized to the WD (FR) or WC (NF) real‐time RT‐PCR values. *gun*,* genomes uncoupled*;*PSA*,* photosystem I* (*PSI*) *subunit*;*FD*,* FERREDOXIN*;*FNR*,* ferredoxin*:*NADP(H) oxidoreductase; PSB*,*PSII subunit; PET*,* cytochrome b*_6_ *f subunit*;*ATPD*,*ATP synthase subunit*;*PDE*,* PIGMENT DEFECTIVE*;*LHC*,* LIGHT HARVESTING CHLOROPHYLL A*/*B BINDING PROTEIN; ELIP*,* EARLY LIGHT*‐*INDUCIBLE PROTEIN*.

To test whether the retrograde signal after an FR pretreatment was the same as that after an NF treatment, we compared gene expression profiles using the 22K Affymetrix ATH1 Arabidopsis microarray in WT (Col‐0) and in *gun1gun5* double mutant seedlings in which GUN signalling is blocked (Fig. 1a). For both data sets there was strong correlation between replicates that was confirmed in correlation plots of all microarray data (Fig. S2). In WT seedlings treated with NF, there was a two‐fold down‐regulation of 761 genes (Fig. 1b; Table S2), which represents *c*. 3% of the genes present on the array. Comparison with other data sets for NF treatment showed a large overlap, with 228 of the 704 NF down‐regulated genes identified by Aluru *et al*. (2009) and 491 of the 1140 genes identified by Koussevitzky *et al*. (2007) represented in this gene cohort, even though these studies were performed in more mature plants and under different experimental conditions. When WT seedlings were grown for 2 d in FR before transfer to WL for 24 h, 442 genes were identified as two‐fold inhibited (Fig. 1b; Table S3) and, as expected, this response showed an almost complete rescue in the *phyA* mutant (Table S3). In total, 1140 different genes showed a two‐fold inhibition of expression in response to either NF or FR pretreatment, but just 63 (6%) were common to both (Fig. 1b; Table S4). This strongly suggests that the retrograde signalling pathways initiated by the two treatments are essentially distinct. Nevertheless, inhibition of both gene cohorts was mitigated in the *gun1gun5* mutant, with 154 (35%) rescued in *gun1gun5* (defined as a 1.5‐fold increase in expression compared with WT) following an FR pretreatment, and 326 (43%) genes rescued after NF treatment (Fig. 1a,b; Tables S2, S3, S5). Evidence that the two treatments target different retrograde signals is also provided by the finding of differences in the predicted intracellular targeting of the proteins encoded by the retrograde‐regulated gene sets (Table S6), and the limited overlap of the gene groups induced by NF and FR pretreatment (Fig. 1a,b; Tables S7, S8, S9). FR pretreatment resulted in a two‐fold induction of 263 genes compared with D‐treated controls of which 192 (73%) were rescued (no induction after FR pretreatment) by the *gun1gun5* mutations (Fig. 1a,b; Table S7). The induction of gene expression was also blocked by the *phyA* mutation (Table S7). NF treatment resulted in a two‐fold induction of 367 genes and just 43 of these were rescued in the *gun1gun5* mutant (Fig. 1a,b; Table S8). Again, the overlap between the two WT gene sets was low, with only 37 (6%) genes induced by both treatments (Fig. 1b; Table S9), further supporting the conclusion that these retrograde responses are distinct.

The different impacts of NF and an FR pretreatment on nuclear gene expression were most clearly apparent when examining genes encoding components of the photosynthetic light reactions (Fig. 1c,d). Of the 55 nuclear‐encoded, photosynthesis‐related genes on the microarray, 47 were more than two‐fold inhibited in NF‐treated seedlings (of which 42 showed a > 1.5‐fold rescue by *gun1gun5*), but only nine were inhibited > 1.5‐fold following an FR pretreatment (Fig. 1c). However, this set of responsive genes encoded at least one member of each of the major photosynthetic complexes (i.e. photosystem I (PSI), PSII, cytochrome *b*_*6*_ *f* and ATP synthase), as well as a representative of each of the *LHCA* and *LHCB* gene families (Fig. 1c). This relationship was confirmed by real‐time PCR analysis of seven photosynthesis‐related genes (Fig. 1d). As the NF experiment was carried out in the presence of sucrose to follow standard protocols, the real‐time PCR experiments were also performed in the absence of sucrose, as sucrose has been shown to be an important regulator of photosynthetic gene expression (Hanson & Smeekens, 2009). The data in Fig. 1(d) demonstrate that the real‐time PCR analysis was consistent with the respective expression profiles determined from the array. In addition, the presence or absence of sucrose was not found to significantly influence the qualitative response to NF.

**Figure 2:**
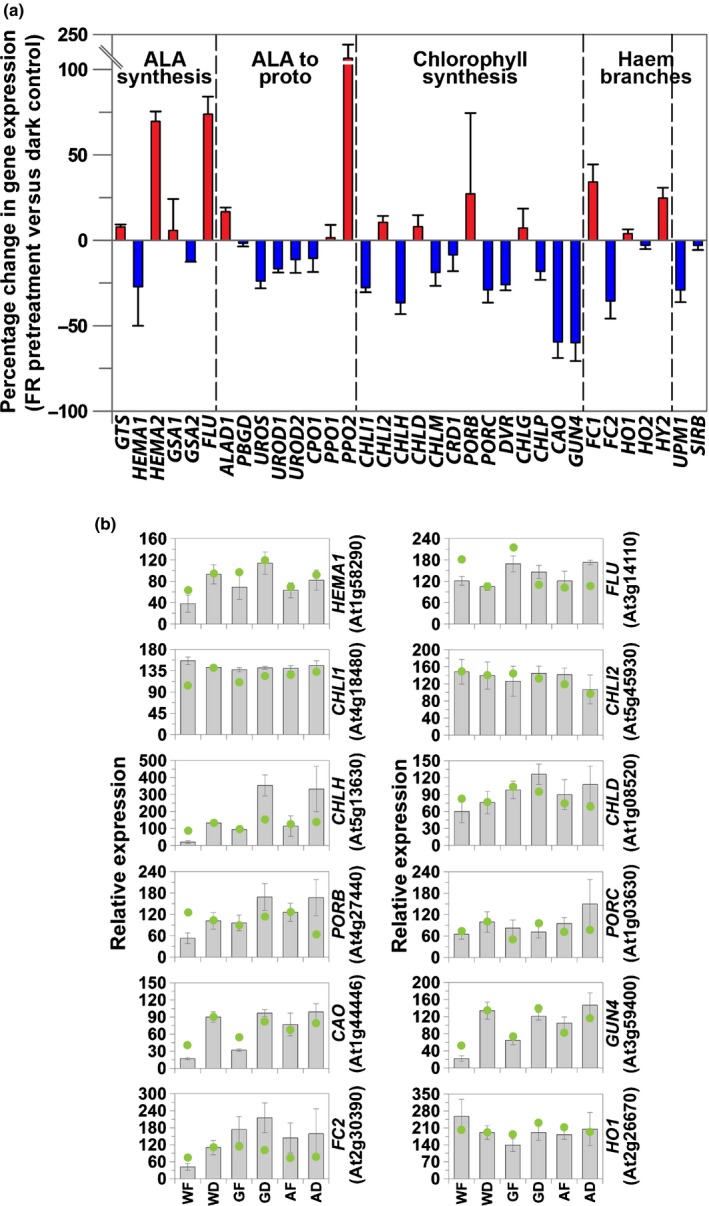
The effect of a far‐red light (FR) pretreatment on expression of *Arabidopsis thaliana* genes encoding enzymes involved in the tetrapyrrole pathway. (a) Microarray analysis of tetrapyrrole pathway genes in wild‐type (WT) seedlings in white light after an FR pretreatment compared with a dark (D) pretreated control, with induced genes represented by red bars and inhibited genes by blue bars. Data shown are the mean and range of two independent experiments. (b) Real‐time reverse transcription−polymerase chain reaction (RT‐PCR) analysis of tetrapyrrole synthesis genes. WT,* gun1gun5* or *phytochrome A* (*phyA*) seedlings were grown with a pretreatment of FR (WF, GF and AF, respectively) or D (WD, GD and AD, respectively). Data shown are the mean ± SE (*n* = 7 independent experiments) and array data are represented by green dots, normalized to the WD real‐time RT‐PCR values. ALA, aminolevulinic acid; *gun*,* genomes uncoupled*;*GTS*,* GLUTAMYL TRNA SYNTHETASE*;*HEMA*,* glutamyl tRNA reductase*;*GSA*,* GLUTAMATE*‐*1*‐*SEMIALDEHYDE 2,1*‐*AMINOMUTASE*;*FLU*,* FLUORESCENT IN BLUE LIGHT*;*ALAD*,*ALA DEHYDRATASE*;*PBGD*,* PORPHOBILINOGEN DEAMINASE*;*UROS*,* UROPORPHYRINOGEN III SYNTHASE*;*UROD*,* UROPORPHYRINOGEN III DECARBOXYLASE*;*CPO*,* COPROPORPHYRINOGEN III OXIDASE*;*PPO*,* PROTOPORPHYRINOGEN IX OXIDASE*;*CHLI*,* magnesium chelatase subunit I*;*CHLH*,* magnesium chelatase subunit H*;*CHLD*,* magnesium chelatase subunit D*;*CHLM*,* S*‐*adenosyl*‐*L*‐*methionine:magnesium protoporphyrinogen IX methyltransferase*;*CRD*,* COPPER RESPONSE DEFECT*;*POR*,* PROTOCHLOROPHYLLIDE OXIDOREDUCTASE*;*DVR*,* DIVINYL REDUCTASE*;*CHLG*,* chlorophyll synthase*;*CHLP*,* geranylgeranyl pyrophosphate reductase*;*CAO*,* CHLOROPHYLL A OXYGENASE*;*FC*,* FERROCHELATASE*;*HO*,* HAEM OXYGENASE*;*HY*,* ELONGATED HYPOCOTYL*;*UPM*,* UROPORPHYRINOGEN III METHYLTRANSFERASE*;*SIR*,* SIROHYDROCHLORIN FERROCHLEATASE*.

Tetrapyrroles have been strongly implicated as signalling molecules in plastid‐to‐nucleus signalling (Strand *et al*., 2003; Woodson *et al*., 2011; Terry & Smith, 2013), and we also examined the impact of an FR pretreatment on the expression of tetrapyrrole synthesis genes in the microarray data set and by real‐time PCR (Fig. 2). We previously demonstrated that NF treatment resulted in a severe and global knockdown in the expression of chlorophyll synthesis genes (Moulin *et al*., 2008). In contrast to the situation on NF, FR pretreatment had a selective effect on tetrapyrrole synthesis, with only a few genes showing an inhibitory response (Fig. 2a). These included *HEMA1*, encoding glutamyl‐tRNA reductase, *CHLH*,* GUN4* and *CHLOROPHYLL A OXYGENASE* (*CAO*), which correspond to a small cohort of key regulatory genes in the pathway (Matsumoto *et al*., 2004; Stephenson & Terry, 2008), and *FC2*, which has also previously been shown to be regulated by light and NF (Singh *et al*., 2002; Moulin *et al*., 2008). To confirm these results, we undertook real‐time PCR on 12 genes of the tetrapyrrole biosynthesis pathway (Fig. 2b). In general, expression was in close agreement with the microarray data and, in particular, the down‐regulation of *HEMA1*,* CHLH, GUN4, CAO* and *FC2* after an FR pretreatment was confirmed. In addition, the FR pretreatment also induced the expression of some tetrapyrrole biosynthesis genes (Fig. 2a) including *GLUTAMYL tRNA SYNTHETASE*, encoding glutamyl‐tRNA synthetase*, HEMA2*,* PROTOPORPHYRINOGEN OXIDASE 2* (*PPO2*) and *FC1*, all of which are associated with nonphotosynthetic haem synthesis. These genes were also induced by NF (Moulin *et al*., 2008), suggesting that haem synthesis for hemoproteins required in response to oxidative stress is protected following both treatments.

### ^1^O_2_ is implicated as the retrograde signal after an FR pretreatment

**Figure 3:**
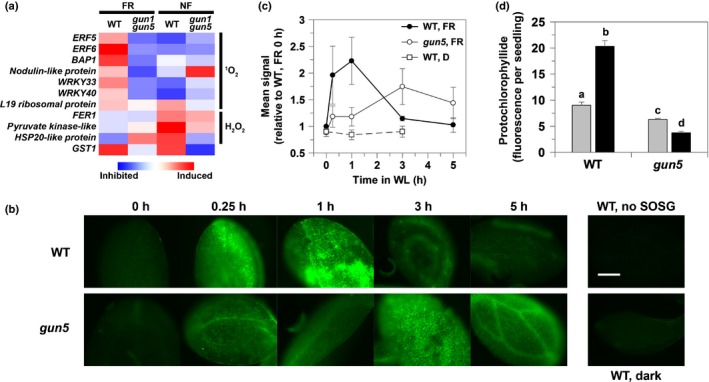
Evidence for the involvement of singlet oxygen (^1^O_2_) in the response to a far‐red light (FR) pretreatment. (a) Heat map depicting microarray analysis of reactive oxygen species marker genes responsive to ^1^O_2_ or hydrogen peroxide (H_2_O_2_) in wild‐type (WT) *Arabidopsis thaliana* seedlings after norflurazon (NF) or an FR pretreatment. Ratios are the mean of two independent experiments. *GLUTATHIONE S‐TRANSFERASE 1* (*GST1*) is an example of a general stress‐responsive gene. (b) Singlet Oxygen Sensor Green (SOSG) staining to detect singlet oxygen generation on transfer of FR‐treated seedlings to white light (WL). Time‐points represent the number of hours after transfer to WL. Controls were FR‐pretreated WT seedlings after 1 h in WL without SOSG (‘WT, no SOSG’), and WT seedlings that did not receive the FR pretreatment, but were maintained in the dark for 3 d (‘WT, dark’). Images shown are representative of three independent biological replicates of WT and *genomes uncoupled 5* (*gun5*) seedlings, and were taken using the same microscope settings and were produced from the same look‐up table; bar, 200 μm. (c) Image intensity analysis for three independent biological replicates of the SOSG staining experiment outlined in (b). Data shown are mean ± SE. (d) Protochlorophyllide content of WT and *gun5* seedlings grown in the dark (grey bars) or after an FR pretreatment (black bars) corresponding to the 0 h time‐point in (b). Data shown are the mean + SE (*n* = 3 independent experiments), with different letters denoting significant differences between group means (*P* < 0.05; Student's *t*‐test). *ERF*,* ETHYLENE RESPONSE FACTOR; BAP*,* BON ASSOCIATION PROTEIN*; WRKY,* WRKY* DNA‐*BINDING PROTEIN*;*FER*,* FERRETIN*;*HSP*,* HEAT SHOCK PROTEIN*;*GST*,* GLUTATHIONE S*‐*TRANSFERASE*.

An FR pretreatment has been shown to lead to an increase in Pchlide (Sperling *et al*., 1997; McCormac & Terry, 2002) and we hypothesized that the signal leading to the retrograde regulation described in this study might be similar to the signal resulting in the induction of ^1^O_2_‐responsive genes in the *flu* mutant of Arabidopsis, which also accumulates high concentrations of Pchlide (Meskauskiene *et al*., 2001; op den Camp *et al*., 2003). As shown in Fig. 3(a), an FR pretreatment did indeed result in the induction of known ^1^O_2_‐responsive genes (op den Camp *et al*., 2003; Danon *et al*., 2005; Lee *et al*., 2007; Kim & Apel, 2013). We also compared our array data after an FR pretreatment to gene expression profiles for the two other well‐characterized ^1^O_2_ signalling systems: the *flu* mutant (op den Camp *et al*., 2003) and the chlorophyll *b*‐less *chlorina1* mutant (Ramel *et al*., 2013). In both these cases, ^1^O_2_‐regulated transcriptomes were determined using plants at the rosette stage and using different time‐points. Nevertheless, there was good overlap of our data with both experimental systems. For example, out of the 70 genes induced specifically by ^1^O_2_ (op den Camp *et al*., 2003), 40 were also induced to some degree in both replicates of the FR pretreatment array. Also, of the 263 genes up‐regulated after an FR pretreatment, 157 were more than two‐fold induced in *flu* after 2 h, with 130 out of 442 down‐regulated genes also down‐regulated two‐fold in *flu* (op den Camp *et al*., 2003). Similarly, 47 of the 263 genes induced by an FR pretreatment were also induced in *chlorina1*, with 80 of the 442 inhibited genes also down‐regulated in *chlorina1* (Ramel *et al*., 2013).

To confirm whether ^1^O_2_ was produced in WL after transfer from FR, we measured ^1^O_2_ production using Singlet Oxygen Sensor Green (SOSG; Flors *et al*., 2006). Fluorescence was rapidly and strongly induced in WT seedlings after transfer to WL from FR, with a fluorescence signal clearly detectable after 15 min and a maximum signal by 1 h (Fig. 3b,c). No induction of ^1^O_2_ was observed in the first 1 h after transfer from dark to WL (Fig. 3b,c). By contrast, the *gun5* mutant, which contains severely reduced Pchlide after an FR treatment (Fig. 3d), showed a much attenuated response with a shallower peak of fluorescence that was also seen far later than in WT seedlings (Fig. 3b,c). The reason for some ^1^O_2_ production in the *gun5* mutant when Pchlide concentrations were low is not clear. One possibility is that the *gun5* mutation, which leads to a decrease in Mg‐chelatase activity, results in an accumulation of the Mg‐chelatase substrate, protoporphyrin IX, which is also a photosensitizer. This could result in some ^1^O_2_ production under longer WL periods as the flux through the tetrapyrrole pathway increases. Consistent with our SOSG results, the dansyl‐based ROS sensor, DanePy, which is specifically quenched by ^1^O_2_ (Hideg *et al*., 2002), also showed fluorescence quenching after an FR pretreatment in WT seedlings, but not in a *phyA* mutant (Fig. S3). NF treatment might also be expected to produce ^1^O_2_ as a result of photo‐excitation of chlorophyll in the absence of carotenoids, as has been observed for light‐grown seedlings treated with NF (Kim & Apel, 2013). However, seedlings treated with NF from germination do not show a ^1^O_2_ response (Kim & Apel, 2013) and no evidence for ^1^O_2_ production was observed here (Figs 3a, S3). By contrast, FR pretreatment did not induce H_2_O_2_‐specific transcripts (op den Camp *et al*., 2003), and these were instead elevated after NF treatment (Fig. 3a).

**Figure 4:**
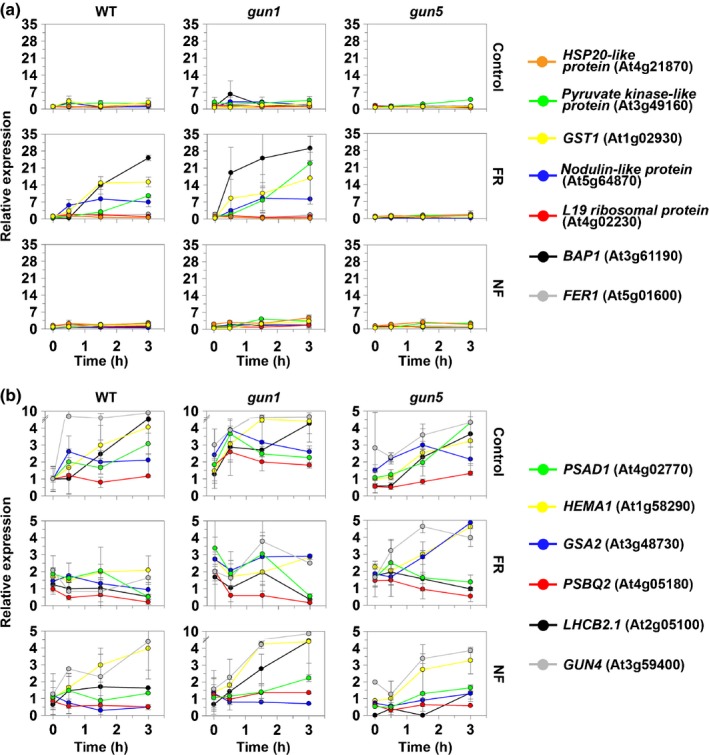
Time‐course of changes in nuclear gene expression in response to norflurazon (NF) and a far‐red light (FR) pretreatment measured by real‐time reverse transcription−polymerase chain reaction (RT‐PCR). Wild‐type (WT), *genomes uncoupled 1* (*gun1*) and *gun5 Arabidopsis thaliana* seedlings were grown for 3 d in the dark (D) in the presence (NF) or absence (control) of NF or grown for 1 d in D followed by a 2‐d FR pretreatment (FR). All samples were then transferred to white light for 3 h. (a) Reactive oxygen species marker genes. (b) Photosynthesis‐/tetrapyrrole‐associated genes. Data shown are the mean ± SE (*n* = 3 independent experiments), normalized to the WT control (*t* = 0) value for each transcript series. *HSP*,* HEAT SHOCK PROTEIN*;*GST*,* GLUTATHIONE* S‐*TRANSFERASE*;*BAP*,* BON ASSOCIATION PROTEIN*;*FER*,* FERRETIN*;*PSA*,* photosystem I (PSI*) *subunit*; HEMA,* glutamyl* tRNA *reductase*; GSA,* GLUTAMATE*‐*1*‐*SEMIALDEHYDE 2,1 AMINOMUTASE*;*PSB*,*PSII SUBUNIT*;*LHCB*,* LIGHT HARVESTING CHLOROPHYLL A*/*B BINDING PROTEIN*.

To investigate how rapidly changes in nuclear gene expression could be observed after an FR pretreatment, we conducted a time course expression profile over 3 h for seven ROS‐responsive genes (Fig. 4a) and six photosynthesis‐related genes (Fig. 4b). The microarray data were obtained with the *gun1gun5* double mutant and therefore to break this response down further we conducted this experiment using the monogenic *gun1* and *gun5* mutants. Upon transfer to WL, the FR‐pretreated WT seedlings displayed a strong and rapid up‐regulation of two ^1^O_2_‐responsive genes, *BON ASSOCIATION PROTEIN 1* (*BAP1*) and *nodulin‐like protein* (Fig. 4a). This induction was abolished in the *gun5* mutant, but was more rapid in the *gun1* mutant than in WT (Fig. 4b), consistent with the more severe effect of an FR pretreatment on the *gun1* mutant (McCormac & Terry, 2004). Although some ^1^O_2_ production was observed in *gun5* (Fig. 3b,c), it was only apparent after 3 h, which may have been too late to induce gene expression in this assay. Control (3 d in the dark before transfer to WL without NF) and NF‐treated seedlings of all lines showed little induction of ROS‐responsive genes over this time course (Fig. 4a). Photosynthesis‐related genes were induced after transfer from the dark to WL. However, in parallel to the rapid induction of ^1^O_2_‐induced genes, expression of photosynthesis‐related genes was strongly inhibited in FR‐pretreated seedlings, with differences observed from control samples after just 30 min in WL in some cases (Figs 4b, S4). Furthermore, the most sensitive transcripts, *GUN4* and *PHOTOSYSTEM II SUBUNIT Q2* (*PSBQ2*), were depleted within 0.5 h in WL to below the levels seen at the time of initial transfer from FR. The rapid response in gene expression was consistent with the induction of ^1^O_2_ observed within 15 min in the SOSG assay (Fig. 3b,c). Again, as seen for induction of ^1^O_2_‐responsive genes, *gun1* mutant seedlings showed an exacerbated inhibitory response to an FR pretreatment over the first 30 min, although *gun1* seedlings had higher levels of expression on transfer to WL (as noted previously; McCormac & Terry, 2004), while the *gun5* mutant completely rescued the early WL response of *GUN4*,* GLUTAMATE‐1‐SEMIALDEHYDE 2,1‐AMINOMUTASE 2* (*GSA2*) and *HEMA1* from inhibition by an FR pretreatment and partially rescued all other genes (Figs 4b, S4). Again, this rescue was consistent with the attenuated production of ^1^O_2_ as shown by SOSG (Fig. 3c). We previously showed the effect of the *gun1gun5* mutations on gene expression after an FR pretreatment and 24 h in WL (Figs 1, 2). To enable a direct comparison with the *gun5* single mutant, we also analysed expression in *gun5* at this time‐point (Fig. S5). Under these conditions, the *gun5* mutant was able to rescue expression to a similar degree to the *gun1gun5* double mutant.

Changes in photosynthetic gene expression after NF treatment were less pronounced than after an FR pretreatment and showed partial rescue in the *gun1* mutant, but not in *gun5,* over this 3‐h period (Fig. 4b). The two retrograde signals can therefore be further distinguished by the relative impact of the *gun1* and *gun5* mutations on the responses.

### Retrograde signalling after an FR pretreatment is partially dependent on *EXECUTER* proteins

**Figure 5:**
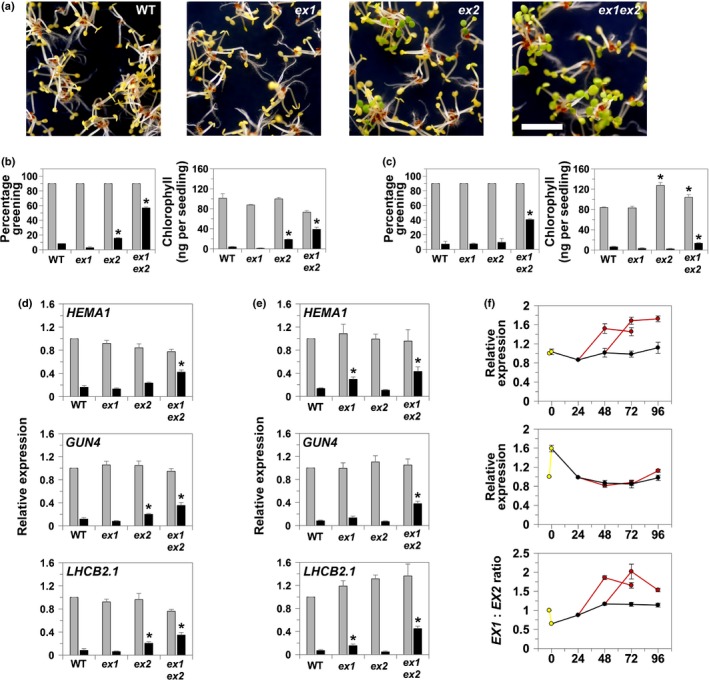
The white light (WL) response of *executer* (*ex*) mutants to a far‐red light (FR) pretreatment. (a) Representative WL phenotype of *ex1*,* ex2* and *ex1ex2 Arabidopsis thaliana* mutant seedlings after an FR pretreatment following 1 d in the dark (D); bar, 5 mm. (b, c) Percentage greening and total chlorophyll content of *ex1*,* ex2* and *ex1ex2* seedlings after an FR (black bars) or dark control (grey bars) pretreatment following an initial incubation in D for (b) 1 d or (c) 2 d. (d, e) Real‐time reverse transcription−polymerase chain reaction (RT‐PCR) analysis of *HEMA1* encoding glutamyl tRNA reductase, *GENOMES UNCOUPLED 4* (*GUN4*) and *LIGHT HARVESTING CHLOROPHYLL A/B BINDING PROTEIN 2.1* (*LHCB2.1*) expression in *ex1*,* ex2* and *ex1ex2* seedlings in WL after an FR (black bars) or D control (grey bars) pretreatment following an initial incubation in D for (d) 1 d or (e) 2 d. For (b–e), data shown are mean + SE (*n* = 4 independent experiments). (f) Quantitative RT‐PCR analysis of *EX1* and *EX2* expression throughout the two different growth regimes used to assess the response of *ex* mutants to an FR pretreatment, with the *EX1*:*EX2* expression ratio also given. Data shown are the mean ± SE (*n* = 3 independent experiments). Line colours correspond to light conditions (yellow, WL; black, D; red, FR). Asterisks denote a significant increase compared to WT (*P* < 0.05; Student's *t*‐test).

To examine further the hypothesis that retrograde signalling after an FR pretreatment is dependent on ^1^O_2_, we examined the effect of the ^1^O_2_ signalling mutants *ex1* (Wagner *et al*., 2004) and *ex2* (Lee *et al*., 2007) on photosynthetic gene expression. Using our standard conditions of 1 d in the dark before the 2‐d FR treatment, the *ex2* mutant showed a partial rescue of greening and this was substantially increased in the *ex1ex2* double mutant (Fig. 5a,b). Rescue was not a result of a reduction in Pchlide concentrations (Fig. S6). Under these conditions, expression of *HEMA1*,* GUN4* and *LHCB2.1* was significantly higher in the *ex2* single mutant and the *ex1ex2* double mutant, with expression restored to *c*. 30–50% in the latter (Fig. 5d). Interestingly, when the dark period was extended to 2 d, we still saw a strong rescue of nuclear gene expression in the *ex1ex2* double mutant, but in this case partial rescue was observed in *ex1* and not *ex2* (Figs 5c,e, S6). A greater role for EX1 compared with EX2 was previously observed for the rescue of gene expression in the *flu* mutant (Lee *et al*., 2007). Analysis of expression data for *EX1* and *EX2* following germination showed that *EX2* is initially elevated compared with *EX1,* with *EX1* expression induced later in development (Fig. 5f). This is consistent with the observed earlier role for EX2.

## Discussion

### NF and FR pretreatments affect different chloroplast‐to‐nucleus signals

The primary biogenic retrograde signal affected by NF treatment is proposed to be a positive signal that is dependent on FC1 enzyme activity in the chloroplast (Woodson *et al*., 2011). Our data suggest that the signal generated by an FR pretreatment is different from the NF signal based on the distinctiveness of the overall gene expression profiles affected by the two treatments, and the relative impact of the *gun1* and *gun5* mutations on the signals. We did, however, observe a small, common set of inhibited genes that showed a significant enrichment for genes encoding predicted chloroplast‐targeted proteins. This included many genes required for the synthesis of the photosynthetic complexes, as well as genes critical for chloroplast development such as *GOLDEN2‐LIKE 2* (*GLK2*; Waters *et al*., 2009). Some overlap in regulation is not surprising as any informational signal affecting chloroplast biogenesis is likely to converge on a few key regulatory genes. The alternative scenario that the common gene set is responding to a unique signal generated under both conditions, with the regulation of condition‐specific genes under the control of separate, additional signals, is far less likely. In this regard, the FR pretreatment resulted in the selective inhibition of just a few chlorophyll synthesis genes, including *HEMA1*,* GUN4*,* CHLH* and *CAO*, which have previously been identified as key regulatory genes in the pathway (Matsumoto *et al*., 2004; Stephenson & Terry, 2008). These results are therefore consistent with the FR pretreatment initiating a targeted and specific down‐regulation of chlorophyll synthesis under these conditions rather than a general inhibition of all the pathway components as seen after NF treatment (Moulin *et al*., 2008). Detailed analysis of gene expression for the four complexes of the photosynthetic light reactions showed a similar pattern. NF treatment caused a strong down‐regulation of almost all photosynthetic genes, while an FR pretreatment only affected a few in each photosystem and just one for ATP synthase (*ATPD* encoding the δ subunit) and the cytochrome *b*_6_ *f* complex (*PLASTOCYANIN 1* (*PETE1*)). It is tempting to speculate that the genes specifically regulated by an FR pretreatment also reflect key regulatory targets for each photosynthetic complex, as seen for the tetrapyrrole pathway.

### The role of ^1^O_2_ in plastid‐to‐nucleus communication

Previous work has unequivocally demonstrated that *flu* mutant seedlings generate a ^1^O_2_ signal on transfer to WL (op den Camp *et al*., 2003; Kim *et al*., 2012), resulting in a severe response leading to seedling death (Danon *et al*., 2005; Kim *et al*., 2012), and attention has focused on the role of ^1^O_2_ signalling in stress (Ramel *et al*., 2013; Zhang *et al*., 2014). Our experimental design, in which light‐regulated photosynthetic genes are induced during the FR treatment, has now allowed us to reveal a previously undiscovered role for ^1^O_2_ signalling as a regulatory retrograde signal during chloroplast biogenesis. By contrast, other studies on ^1^O_2_ signalling have generally not been conducted during the biogenic phase of chloroplast development. The proposed ^1^O_2_ signal works rapidly to inhibit photosynthetic gene expression within 30 min and, in response to moderate increases in chlorophyll precursors that might occur in nature (as compared with the severe conditions of a *flu* mutation or FR pretreatment), would produce an acclimatory response that would serve to modulate chlorophyll synthesis to achieve an optimal synthesis rate under challenging environmental conditions. Under more severe conditions, ^1^O_2_ production results in chloroplast degradation via a ubiquitin‐mediated pathway (Woodson *et al*., 2015) and ultimately cell death (Danon *et al*., 2005; Kim *et al*., 2012).

One study that is potentially similar to ours investigated the phytochrome regulatory mutants *phytochrome interacting factor 1* (*pif1*) and *pif3*. These mutants show elevated Pchlide as the PIF1 and PIF3 proteins are required to repress chloroplast development in darkness (Huq *et al*., 2004; Shin *et al*., 2009; Stephenson *et al*., 2009). In fact, dark‐grown *pif* mutants behave in a similar way to the situation we observe after an FR pretreatment, with activation of phytochrome responses, but insufficient light for photoconversion of Pchlide. Consistent with this, *pif1* and *pif3* mutants also produce ^1^O_2_ on transfer to WL (Chen *et al*., 2013), with significant overlap of the gene sets regulated by the two treatments: 137 of the 263 genes induced by an FR pretreatment were also induced in *pif1* seedlings and 115 of the 442 genes inhibited by an FR pretreatment were also inhibited in *pif1*. Interestingly, the rice mutant *faded green leaf*, which lacks PROTOCHLOROPHYLLIDE OXIDOREDUCTASE B, also accumulates ^1^O_2_ in light‐grown plants and showed a strong down‐regulation of photosynthesis‐related genes including *HEMA1, CHLH, CAO* and *LHCB1* (Sakuraba *et al*., 2013).

**Figure 6:**
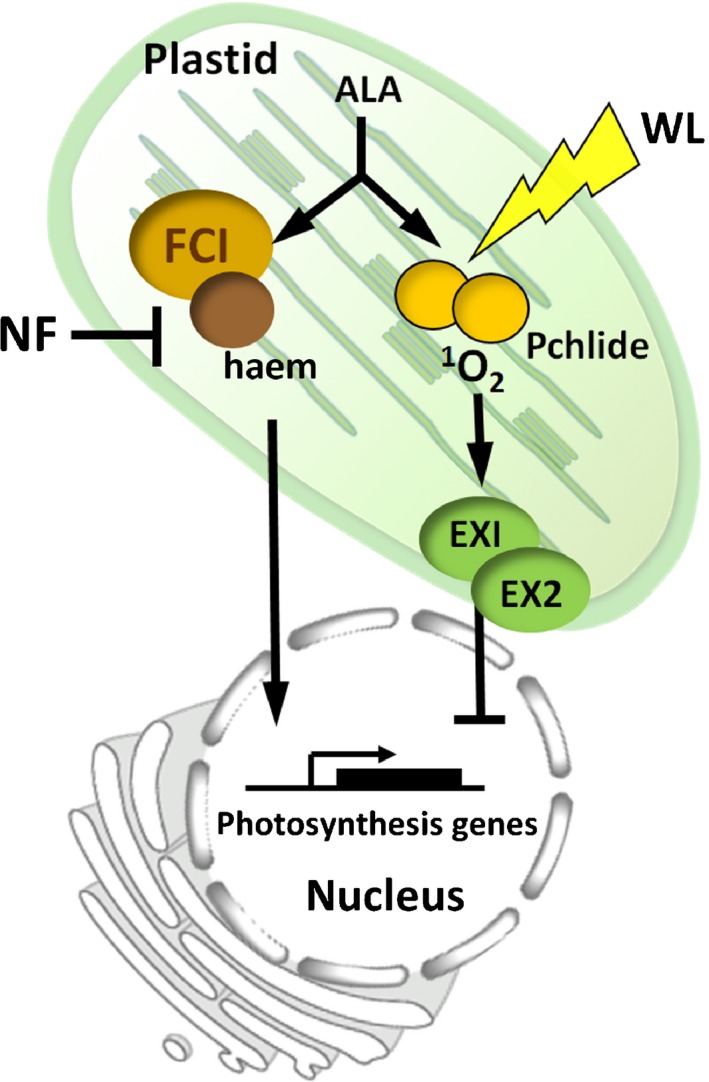
A model for tetrapyrrole regulation of nuclear gene expression. Both norflurazon (NF) and a far‐red light (FR) pretreatment result in inhibition of nuclear gene expression by activating different signalling pathways. NF treatment inhibits a ferrochelatase1‐dependent haem‐related positive signal that promotes photosynthetic gene expression (Woodson *et al*., 2011). After an FR pretreatment, there is an accumulation of protochlorophyllide, which generates a singlet oxygen (^1^O_2_) signal on transfer to white light (WL). This results in the activation of ^1^O_2_ marker genes and an inhibition of specific photosynthesis‐related genes in a signalling pathway that is partially dependent on EXECUTER1 (EX1) and EX2. ALA, aminolevulinic acid.

Our data therefore support a new model in which two tetrapyrrole‐related signals regulate photosynthesis‐related nuclear genes (Fig. 6). NF treatment inhibits the synthesis of a specific FC1‐dependent haem pool that would normally promote photosynthesis‐related nuclear gene expression (Woodson *et al*., 2011), most probably by permitting normal light induction of these genes (Ruckle *et al*., 2007; Martin *et al*., 2016). This signal measures the general requirement for plastid proteins as a function of the number and developmental status of the plastids and has a broad effect on the expression of nuclear photosynthetic genes. However, under conditions in which tetrapyrrole synthesis is elevated and synthesis of potentially damaging chlorophyll intermediates might compromise seedling survival, there is a rapid down‐regulation of selected key regulatory genes to prevent overaccumulation of tetrapyrroles and repress chloroplast development. Such conditions might include severe shade (i.e. similar conditions to those used in this study), nutritional deficiencies such as low metal availability, or the presence of contaminants in the soil that alter tetrapyrrole flux. Our data suggest that this inhibitory signal is a ^1^O_2_‐mediated signal generated by direct excitation of free chlorophyll intermediates (Terry & Smith, 2013). Although the signal analysed in this study is primarily generated by Pchlide overaccumulation, in principle any porphyrin (or chlorin) could generate such a signal (Redmond & Gamlin, 1999), including Mg‐protoporphyrin IX, and this observation may reconcile some discrepancies in the literature (Strand *et al*., 2003; Zhang *et al*., 2011; Kindgren *et al*., 2012). The current study has focused on the situation during chloroplast biogenesis in which there is a large increase in flux through the tetrapyrrole pathway that brings new dangers to a developing seedling. In the future, it will be interesting to test whether such a signalling pathway could operate in mature plants. At this later developmental stage, the major source of ^1^O_2_ is the excitation of chlorophyll molecules in the light‐harvesting antenna complexes and the photosystem II (PSII) reaction centres (Hideg *et al*., 1998; Triantaphylidès & Havaux, 2009). Overexcitation of these complexes would result in increased ^1^O_2_ production and photoinhibition, and an inhibition of the tetrapyrrole pathway via ^1^O_2_ signalling could form part of an integrated response to this problem. It is also noteworthy that the PSII genes *PSBQ2* and *PSAD1* were both rapidly inhibited in this study, suggesting that regulation of photosystem components is an important function of this signalling pathway. Such a pathway would serve as part of the operational chloroplast signalling network conveying the impact of the environment on chloroplast status to the rest of the cell (Pogson *et al*., 2008).

In our study, retrograde regulation of photosynthetic gene expression by ^1^O_2_ signalling was only partially mediated by EX1 and EX2, suggesting that other pathways may also be involved. One possibility is the carotenoid oxidation product β‐cyclocitral (Ramel *et al*., 2012), which functions independently of the EX proteins and mediates inhibition of some photosynthetic genes, such as *GUN4*,* CAO* and *FC2,* that are the most repressed following an FR pretreatment (Ramel *et al*., 2013). There are also a number of other possibilities for signalling molecules, including dihydroactinidiolide, another secondary metabolite of β‐carotene, which is EX‐independent (Shumbe *et al*., 2014), and products of EX‐dependent enzymatic lipid peroxidation (Przybyla *et al*., 2008). The zinc finger protein METHYLENE BLUE SENSITIVITY1 has also been proposed to play a role in ^1^O_2_ signalling (Shao *et al*., 2013). Understanding the relationship between these different ^1^O_2_ signalling pathways will be key to elucidating ^1^O_2_‐mediated retrograde signalling of photosynthetic gene expression in the future.

In summary, our data identify the primary consequence of an FR pretreatment as the production of ^1^O_2_, which leads to the inhibition of expression of nuclear‐encoded chloroplast proteins via a chloroplast‐generated signal that is distinct from that observed after NF treatment. The *flu, chlorina* and *fc2* mutants have all proved invaluable for studying the cellular consequences of ^1^O_2_ production (Ramel *et al*., 2013; Zhang *et al*., 2014; Woodson *et al*., 2015) and we believe that an FR pretreatment may prove to be equally useful for investigating ^1^O_2_ responses as it allows the controlled and noninvasive induction of chloroplast‐localized ^1^O_2_ in the absence of any requirement for a specific mutant background. This should permit further dissection of the acclimatory and stress‐responsive roles attributed to ^1^O_2_ signalling in plants.

## Author contributions

M.T.P. and A.C.M. designed and performed experiments, analysed data and contributed to writing the manuscript. A.G.S. analysed data and contributed to writing the manuscript. M.J.T. designed experiments, analysed data and wrote the manuscript.

## Supporting information

Please note: Wiley Blackwell are not responsible for the content or functionality of any Supporting Information supplied by the authors. Any queries (other than missing material) should be directed to the *New Phytologist* Central Office.

**Fig. S1** Changes in *GUN4* and *HEMA1* expression in response to a far‐red light pretreatment and a norflurazon treatment assessed with different real‐time RT‐PCR reference genes.
**Fig. S2** Correlation plots of raw expression values from the microarray data set.
**Fig. S3** Detection of singlet oxygen production via quenching of DanePy fluorescence after a far‐red light pretreatment and a norflurazon treatment.
**Fig. S4** Time‐course of changes in photosynthetic gene expression in response to a far‐red pretreatment measured by real‐time RT‐PCR.
**Fig. S5** Rescue of nuclear gene expression in *gun5* after a far‐red pretreatment.
**Fig. S6** Response of *ex* mutants to a far‐red pretreatment.

**Table S1** List of genes referred to in this paper with real‐time PCR primer sequences given (where used)

**Table S2** List of 761 genes inhibited at least two‐fold in WL in WT after NF treatment

**Table S3** List of 442 genes inhibited at least two‐fold in WL in WT after FR pretreatment

**Table S4** List of 63 genes inhibited at least two‐fold in WT by both FR and NF treatments

**Table S5** *gun1gun5* rescue of genes differentially expressed in WT

**Table S6** Predicted localization of protein products of differentially expressed genes identified through microarray analysis

**Table S7** List of 263 genes induced at least two‐fold in WL in WT after an FR pretreatment

**Table S8** List of 367 genes induced at least two‐fold in WL in WT after NF treatment

**Table S9** List of 37 genes induced at least two‐fold in WT by both FR and NF treatments
