# Supplementary material for: Singlet oxygen initiates a plastid signal controlling photosynthetic gene expression
Source: New Phytol. 2016 Oct 13;213(3):1168–80. doi: 10.1111/nph.14223 (PMC5244666; doi:10.1111/nph.14223)
Supplement: Supplementary file 1 — Fig. S1 Changes in GUN4 and HEMA1 expression in response to a far‐red light pretreatment and a norflurazon treatment assessed with different real‐time RT‐PCR reference genes. Fig. S2 Correlation plots of raw expression values from the microarray data set. Fig. S3 Detection of singlet oxygen production via quenching of DanePy fluorescence after a far‐red light pretreatment and a norflurazon treatment. Fig. S4 Time‐course of changes in photosynthetic gene expression in response to a far‐red pretreatment measured by real‐time RT‐PCR. Fig. S5 Rescue of nuclear gene expression in gun5 after a far‐red pretreatment. Fig. S6 Response of ex mutants to a far‐red pretreatment. [file NPH-213-1168-s001.pdf]

New Phytologist

New Phytologist **Supporting Information Figs S1–S6**

Article title: Singlet oxygen initiates a plastid signal controlling photosynthetic gene expression

Authors: Mike T. Page, Alex C. McCormac, Alison G. Smith and Matthew J. Terry

Article acceptance date: 19 August 2016

The following Supporting Information is available for this article:

Fig. S1 Changes in *GUN4* and *HEMA1* expression in response to a far-red light pretreatment and a Norflurazon treatment assessed with different real-time RT-PCR reference genes.

Fig. S2 Correlation plots of raw expression values from the microarray data set.

Fig. S3 Detection of singlet oxygen production *via* quenching of DanePy fluorescence after a far-red light pretreatment and a Norflurazon treatment.

Fig. S4 Time-course of changes in photosynthetic gene expression in response to a far-red pretreatment measured by real-time RT-PCR.

Fig. S5 Rescue of nuclear gene expression in *gun5* after a far-red pretreatment.

Fig. S6 Response of *ex* mutants to a far-red pretreatment.

Table S1 List of genes referred to in this manuscript with real-time PCR primer sequences given (separate Excel file)

Table S2 List of 761 genes inhibited at least two-fold in WL in WT after NF treatment (separate Excel file)

Table S3 List of 442 genes inhibited at least two-fold in WL in WT after FR pretreatment (separate Excel file)

Table S4 List of 63 genes inhibited at least two-fold in WT by both FR and NF treatments (separate Excel file)

Table S5 *gun1gun5* rescue of genes differentially expressed in WT (separate Excel file)

Table S6 Predicted localisation of protein products of differentially expressed genes identified through microarray analysis (separate Excel file)

Table S7 List of 263 genes induced at least two-fold in WL in WT after a FR pretreatment (separate Excel file)

Table S8 List of 367 genes induced at least two-fold in WL in WT after NF treatment (separate Excel file)

Table S9 List of 37 genes induced at least two-fold in WT by both FR and NF treatments (separate Excel file)

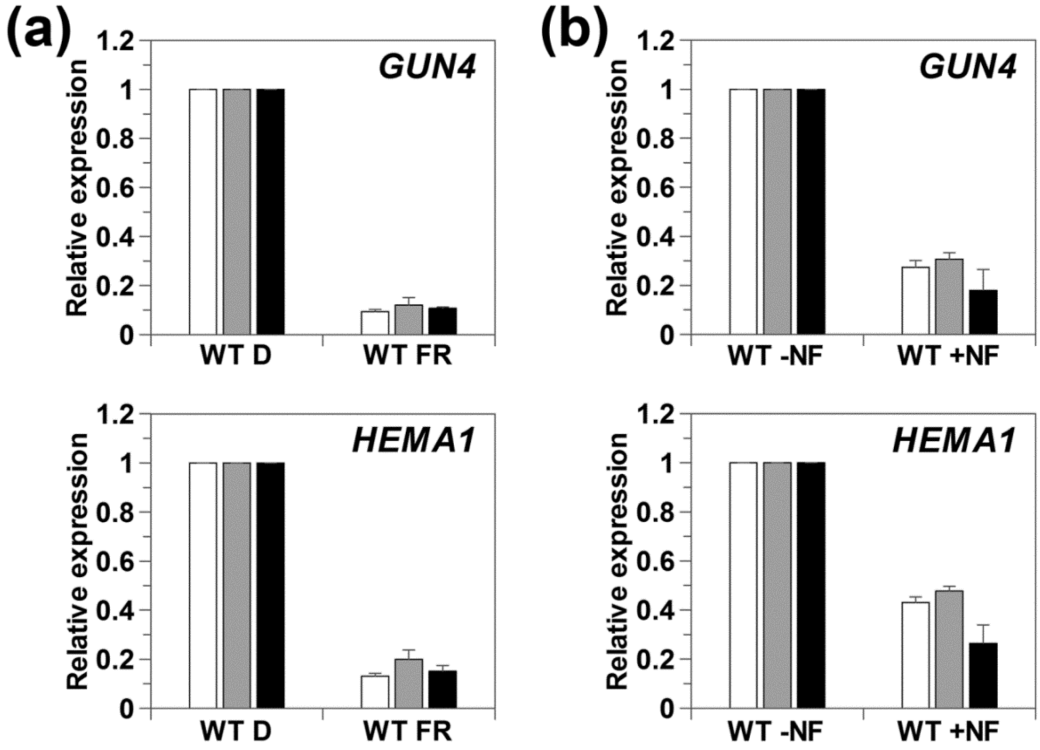

Fig. S1 Changes in *GUN4* and *HEMA1* expression in response to a far-red light (FR) pretreatment and a Norflurazon (NF) treatment assessed with different real-time RT-PCR reference genes. (a) For the FR treatment, wild-type (WT) *Arabidopsis thaliana* seedlings were incubated for 2 d in the dark (D), followed by a 2 d FR pretreatment (WT FR) or kept in D for 2 d (WT D), and then transferred to white light (WL) for 1 d. (b) For the NF treatment, seedlings were grown for 7 d in low WL (25 µmol m^{-2} s^{-1}) in the presence (WT +NF) or absence (WT –NF) of 5 µM NF (WT +NF). Relative expression of *GUN4* and *HEMA1* was normalised to *ADF2* (white bars), *YLS8* (grey bars) or *18S* (black bars). Data shown are the mean + SE (*n* = 3 independent experiments).

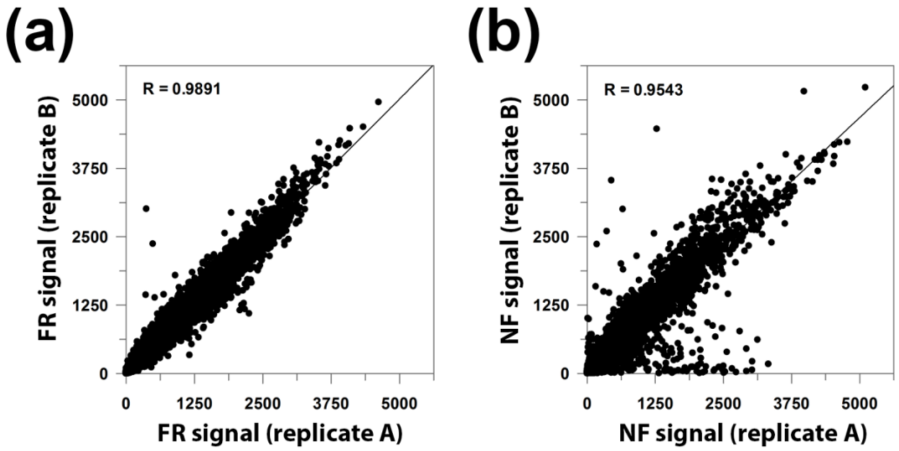

Fig. S2 Correlation plots of raw expression values from the microarray data set. Gene expression signal values for all genes on the Affymetrix 22k ATH1 microarray were plotted for the two replicates of (a) the far-red light (FR) pretreatment and (b) Norflurazon (NF) data sets.

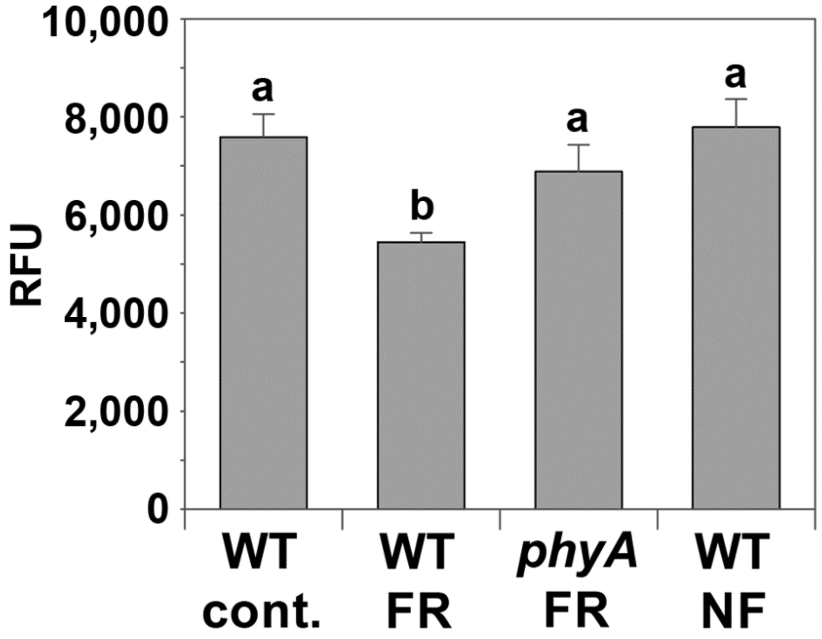

Fig. S3 Detection of singlet oxygen production *via* quenching of DanePy fluorescence after a far-red light (FR) pretreatment and a Norflurazon (NF) treatment. Data shown are the mean (in relative fluorescence units, RFU) ± SE (n = 4 from two independent experiments). Different letters (a, b) denote significant differences between group means (P < 0.05).

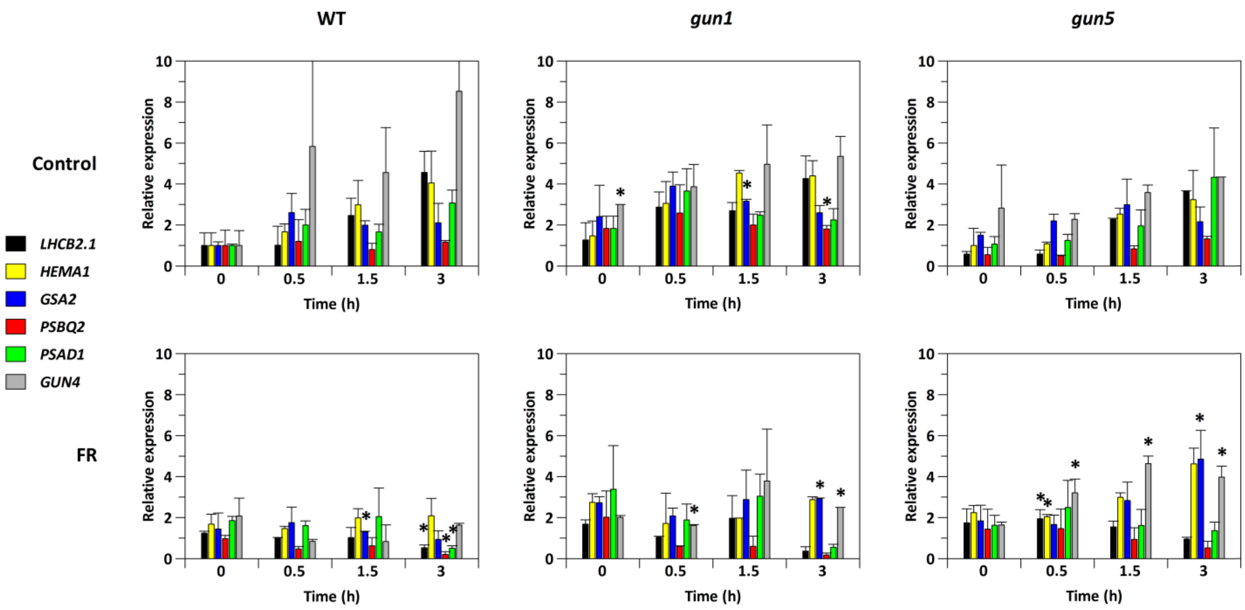

Fig. S4 Time-course of changes in photosynthetic gene expression in response to a far-red light (FR) pretreatment measured by real-time RT-PCR. Data replotted from the control and FR line graphs in Fig. 4(b). Data shown are the mean ± SE (*n* = 3 independent experiments). Asterisks in the wild-type (WT) FR panel indicate a significant down-regulation of expression in WT after a FR pretreatment, while asterisks in the *gun1* and *gun5* panels indicate a significant difference of gene expression vs WT for the control (upper two panels) or FR treated (lower two panels) seedlings ($P < 0.05$).

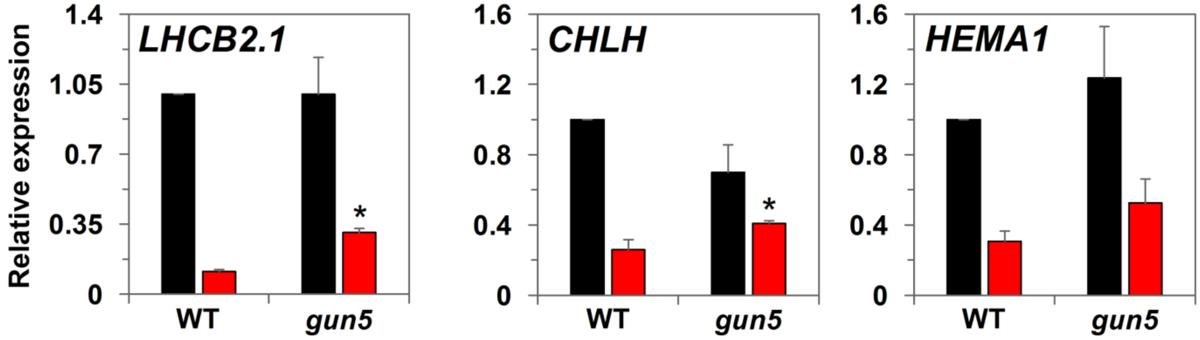

Fig. S5 Rescue of nuclear gene expression in *gun5* after a far-red light (FR) pretreatment. Wild-type (WT) and *gun5 Arabidopsis thaliana* seedlings were incubated for 2 d in the dark (D), followed by a 2 d FR pretreatment (red bars) or kept in D for 2 d (black bars), then transferred to white light for 1 d. The expression of *LHCB2.1*, *CHLH* and *HEMA1* was assessed by real-time RT-PCR. Data represents the mean + SE (*n* = 3 independent experiments). Asterisks denote significant differences vs WT (Student's *t*-test, *P* < 0.05).

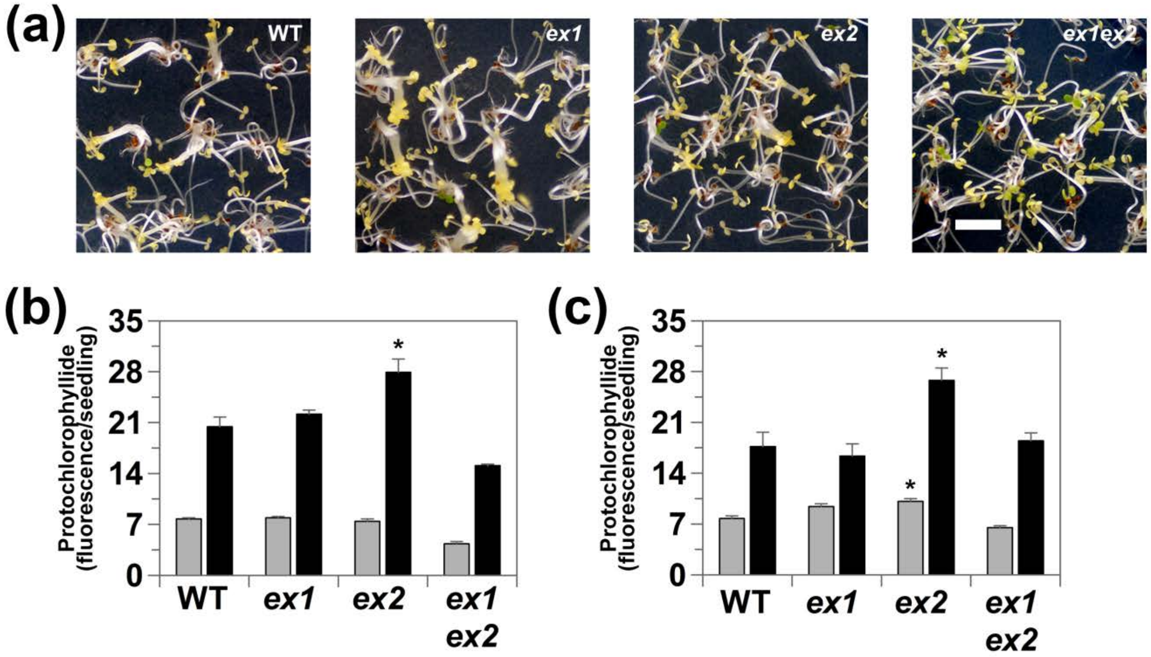

Fig. S6 Response of *ex* mutants to a far-red light (FR) pretreatment. (a) Representative white light phenotype of *ex1*, *ex2* and *ex1ex2* mutant *Arabidopsis thaliana* seedlings after a FR pretreatment following 2 d dark (D), bar, 5 mm. (b, c) Protochlorophyllide content of *ex1*, *ex2* and *ex1ex2* seedlings after a FR (black bars) or D control (grey bars) pretreatment following an initial (b) 1 d D or (c) 2 d D incubation. Data shown are the mean ± SE (n = 3 independent experiments). Asterisks denote significant differences vs WT for the dark or FR pretreatments (Student's t-test, $P < 0.05$).
