# Supplementary material for: Singlet oxygen initiates a plastid signal controlling photosynthetic gene expression
Source: New Phytol. 2016 Oct 13;213(3):1168–80. doi: 10.1111/nph.14223 (PMC5244666; doi:10.1111/nph.14223)
Supplement: Supplementary file 2 — Table S1 List of genes referred to in this paper with real‐time PCR primer sequences given (where used) [file NPH-213-1168-s002.pdf]

Supporting Information Table S1 List of genes referred to in this manuscript, with real-time PCR primer sequences given (where used)

Gene Name	AGI	Sense Primer (5' -> 3')	Antisense Primer (5' -> 3')
18S	At3g41768	GTTGCTTATAGGACTCCGCTG	CCTTACTATGTCTGGACCTGGT
PSAD1	At4g02770	GTCTCCGATCTTCGCTGGAAG	CTTGGATCTAAGCCTTGTCC
PSAD2	At1g03130	GAAGCTCCAGTTGGATTCACTC	CCGATGGATCTCATGTTTAGTCC
FNR2	At1g20020	GGCGACTACCATGAATGCTG	CAGGAGCATCATCAGCTGTG
PSBQ2	At4g05180	GCTCAGCAGAACGTGTCAG	GTAACGGAGGTACGATGCTC
ATPD	At4g09650	GCTACGCGATGGCATTAGCAG	CTCCTTAACGATCTCCGTCACG
LHCB4.2	At3g08940	GAGCTGATTCACGGTCGGTG	GGTCAGACGCTAGTCCTAACG
ELIP2	At4g14690	CAAGAGGATTGCTCCGGTAG	GAACCATCGATGCCAACGTC
ACTIN2	At3g18780	GGTAACATTGTGCTCAGTGGTGG	CTCGGCCTTGGAGATCCACATC
Pyruvate kinase-like protein	At3g49160	CCGTTCTTCGACAAGAGTTGA	CATCACACAGCTCGCCCT
HSP20-like protein	At4g21870	CATGTTCGTTGGTCTCAGTCT	GCAGCTCTAGCAAGAAGTTGA
GST1	At1g02930	GAGCCTTTCATCCTTCGCAA	GTGAAGTGGTCAGAAGCCAA
Nodulin-like protein	At5g64870	GGTGAAGACTGAAGTCAAAGTGT	CTGCGCCTAAGAGGGTCT
L19 ribosomal protein	At4g02230	CATCATCAGGAAGCCAACGA	CTAGCCTTCTCAGCCTTGGA
BAP1	At3g61190	GGAGATTGATTTGAGATCAGCA	CTCGGCCTCCACAAACCA
FER1	At5g01600	CAACGTCTCCTATGTGTACCAT	CATCTGGTCGAAATGCCAA
HEMA1	At1g58290	CAAGAACTCTGCAGCTGATC	CCATTCAGCTTCAGGTATAGC
GSA2	At3g48730	CTTCGTCGTCCAACCGT	CAATGTATTCATTTCCATCAATGTCT
LHCB2.1	At2g05100	CTCCGCAAGGTTGGTGTATC	CGGTTAGGTAGGACGGTGTATT
GUN4	At3g59400	CTCCATTGCCAATCTCAC	CCGAATCTACCATCACTGTG
ADF2	At3g46000	CGATTTCGACTTTGTCACTGC	TCATCTTGTCTCTCACTTTGGC
YLS8	At5g08290	GCTGAAATATCCCGTGAACTG	AATGGAGAACAACCGAAACAG
EX1	At4g33630	CTACTGCTACTAATGATGCTGTTG	GACATCTTCCGAAATACCAGAC
EX2	At1g27510	CGTTGGAACCGTCACTTCTC	CAGCGACAGCATCATCAACA
FLU	At3g14110	GTGACAAGTCTCGAGCTCCAG	CAAGAGGTGTAGCCATCTGAAG
CHLI1	At4g18480	CGGTTATGAATGTAGCCACTG	CTTGCCCTACTATAGCTGC
CHLI2	At5g45930	CCCATCTCTGCTTCGGACCAG	CTCCCATTATCATCACACCACC
CHLH/GUN5	At5g13630	CTGGTCGTGACCCTAGAACAG	GATTGCCAGCTTCTTCTCTG
CHLD	At1g08520	CAGAATTGCCTCGTGCCGCTTC	CATCTGGACAAGCTGGGTCAG
PORB	At4g27440	GGTGCCTCCATTACCGAC	CACGTTCCATTTCCCTGTC
PORC	At1g03630	CCACCCTGTTGACCATCAAG	CAGATCTCGCTGCCTTCTCG
CAO	At1g44446	CTTGGATTGGCGTGCTCG	CGGAACCGGACCAGAAACATTC
FC2	At2g30390	GCTACTTCATCAAACCGGCTTC	GCATCAGTTATGTGGCGAA
HO1/GUN2	At2g26670	GGTTTGTGGCTATGAGACTTCAT	CTGGAATCTCGTAACCTTGT
ERF5	At5g47230		
ERF6	At4g17490		
WRKY33	At2g38470		
WRKY40	At1g80840		
LHCB1.2	At1g29910		
HY2/GUN3	At3g09150		
FC1	At5g26030		
GLK2	At5g44190		
HEMA2	At1g09940		
PPO2	At5g14220		
PETE1	At1g76100		
MBS1	At3g02790		
