# Supplementary material for: Singlet oxygen initiates a plastid signal controlling photosynthetic gene expression
Source: New Phytol. 2016 Oct 13;213(3):1168–80. doi: 10.1111/nph.14223 (PMC5244666; doi:10.1111/nph.14223)
Supplement: Supplementary file 6 — Table S5 gun1gun5 rescue of genes differentially expressed in WT [file NPH-213-1168-s006.pdf]

Supporting Information Table S5 *gun1gun5* rescue of genes differentially expressed in WT

Gene Set	Number of Genes	Number of Genes Rescued at least 1.5-fold in *gun1,5* (%)
**FR Inhibited**	**442**	**154 (34.8%)**
Plastid Localised[a]	15	7 (46.7%)[b] (4.5%)[c]
**FR Induced**	**263**	**192 (73.0%)**
Plastid Localised[a]	10	8 (80.0%)[b] (4.2%)[c]
**NF Inhibited**	**761**	**326 (42.8%)**
Plastid Localised[a]	260	167 (64.2%)[b] (51.2%)[c]
**NF Induced**	**367**	**43 (11.7%)**
Plastid Localised[a]	26	3 (11.5%)[b] (7.0%)[c]
**NF & FR Inhibited**	**63**	**10 (15.9%)**
**NF & FR Induced**	**37**	**1 (2.7%)**

[a]Localisation predictions taken from the TAIR database (www.arabidopsis.org).
[b]Percentage of plastid-localised genes in each set that are rescued in the *gun1gun5* double mutant.
[c]Percentage of genes rescued in the *gun1gun5* double mutant in each set that are plastid-localised.
