# Supplementary material for: Singlet oxygen initiates a plastid signal controlling photosynthetic gene expression
Source: New Phytol. 2016 Oct 13;213(3):1168–80. doi: 10.1111/nph.14223 (PMC5244666; doi:10.1111/nph.14223)
Supplement: Supplementary file 7 — Table S6 Predicted localization of protein products of differentially expressed genes identified through microarray analysis [file NPH-213-1168-s007.pdf]

New Phytologist

Supporting Information Table S6 Predicted localisation of protein products of differentially expressed genes identified through microarray analysis of FR and NF treated WT seedlings

Gene set	Total number of genes	Plastid localisation (%)	Mitochondrion localisation (%)	Secretory pathway localisation (%)	Other (%)
FR Inhibited	442	15 (3.4%)	2 (0.5%)	173 (39.1%)	252 (57.0%)
FR Induced	263	10 (3.8%)	2 (0.8%)	57 (21.7%)	194 (73.7%)
NF Inhibited	761	260 (34.2%)	5 (0.7%)	109 (14.3%)	387 (50.8%)
NF Induced	367	26 (7.1%)	10 (2.7%)	55 (15.0%)	276 (75.2%)
FR & NF Inhibited	63	6 (9.5%)	0 (0.0%)	21 (33.3%)	36 (57.2%)
FR & NF Induced	37	1 (2.7%)	0 (0.0%)	5 (13.5%)	31 (83.8%)

Localisation was determined using the TAIR database (www.arabidopsis.org).
